# Diets Containing Defatted Black Soldier Fly Larvae Meal Improve Intestinal Morphology and Antioxidant Capacity in Wenchang Chickens

**DOI:** 10.3390/metabo16100767

**Published:** 2026-10-12

**Authors:** Qingchao Yang, Ruiying Bao, Liangmin Huang, Xingke Wang, Yang Zhang, Qingying Cai, Zeru Peng, Huiyu Shi, Haiwen Zhang, Xiaochun Li, Xuemei Wang

**Affiliations:** Animal Nutrition and Feed Laboratory, School of Tropical Agriculture and Forestry, Hainan University, Danzhou 571737, China; 24220951330003@hainanu.edu.cn (Q.Y.); 23110710000036@hainanu.edu.cn (R.B.); mlh9809@163.com (L.H.); xkwang28@hainanu.edu.cn (X.W.); 25210905000015@hainanu.edu.cn (Y.Z.); qingyingcai9@163.com (Q.C.); 22210905000001@hainanu.edu.cn (Z.P.); 993978@hainanu.edu.cn (H.S.); hwzhang@hainanu.edu.cn (H.Z.); 990777@hainanu.edu.cn (X.L.)

**Keywords:** defatted black soldier fly larvae meal, Wenchang chickens, antioxidant capacity, ileal morphology, cecal microbiota

## Abstract

**Background/Objectives**: This study evaluated the effects of dietary defatted black soldier fly (*Hermetia illucens*) larvae meal (DBSFLM) as a partial replacement of traditional dietary protein sources on growth performance, carcass traits, meat quality, serum biochemical and antioxidant-related parameters, ileal morphology, and cecal microbiota in Wenchang chickens. **Methods**: Ninety healthy 55-day-old male chickens were randomly assigned to three dietary treatments with six replicates of five birds each: a basal diet without DBSFLM (H0), or diets containing 3% (H3) or 5% (H5) DBSFLM for 26 days. **Results**: Average daily feed intake, average daily gain, and feed conversion ratio did not differ significantly among treatments (*p* > 0.05). Abdominal fat percentage was higher in H5 than in H0 (*p* < 0.05), whereas other measured carcass traits were unaffected. No significant differences were observed in the measured meat quality parameters, including pH, drip loss, cooking yield, shear force, and meat color (*p* > 0.05). Dietary DBSFLM increased serum glutathione peroxidase activity and decreased malondialdehyde concentration, while serum creatinine concentration decreased in both DBSFLM groups (*p* < 0.05). DBSFLM also decreased ileal crypt depth and increased the villus height-to-crypt depth ratio (*p* < 0.05). Changes in the relative abundances of Firmicutes_A, Proteobacteria, *Akkermansia*, Butyricicoccaceae, and *Agathobaculum* were observed in cecal microbiota. **Conclusions**: Overall, partial replacement of traditional dietary protein sources with 3–5% DBSFLM was associated with changes in selected serum parameters, ileal morphology, and cecal microbial composition, without significant differences in growth performance or most measured meat quality traits.

## 1. Introduction

As the global livestock industry continues to grow, limited feed resources and escalating production expenses have emerged as key constraints on the sustainable development of poultry production. Traditional protein feed ingredients are not only subject to considerable price fluctuations, but their production processes are also associated with land resource consumption, environmental pollution, and greenhouse gas emissions [1,2]. Therefore, the development of efficient, safe, and sustainable alternative protein feed resources has become an important direction in current animal nutrition and feed science research [1,3]. Insect protein has gradually gained attention as an important research focus for replacing conventional protein sources due to its high feed conversion efficiency, low environmental footprint, and favorable nutritional value [4,5,6,7,8].

Black soldier fly (*Hermetia illucens*) larvae, as one of the most extensively studied insect resources in recent years, are characterized by rapid growth, high reproductive capacity, and the ability to efficiently convert organic wastes, and are considered a novel feed resource with both ecological benefits and nutritional value [9,10]. The larvae represent a valuable protein source due to their elevated crude protein level, balanced amino acid profile, and abundance of biologically active compounds such as medium-chain fatty acids, antimicrobial peptides, and chitin, demonstrating considerable application potential in the field of animal nutrition [11,12,13]. Evidence suggests that dietary inclusion of black soldier fly larvae meal (BSFLM) may reduce reliance on soybean meal and fish meal while exerting beneficial effects on animal health through modulation of the gut microbiome, stimulation of immunity, and enhancement in antioxidant capacity [14,15,16].

In recent years, the use of BSFLM in poultry feeding has attracted considerable research interest. Relevant research has demonstrated that the inclusion of insect protein ingredients in poultry diets can improve growth performance, feed utilization efficiency, and some intestinal health indicators [17,18,19]. To further enhance protein concentration and product stability, BSFLM can be defatted. Defatted black soldier fly larvae meal (DBSFLM) increases protein content while reducing lipid-related digestive and metabolic burdens in poultry, and retains favorable amino acid digestibility and metabolizable energy, making it a suitable alternative protein ingredient [6,20]. Dabbou et al. [7] found that dietary supplementation with an appropriate amount of DBSFLM improved the growth performance and feed intake of broilers, but an excessively high inclusion level might negatively affect intestinal morphology. Additional studies have indicated that BSFLM can enhance protein digestibility, improve feed conversion efficiency, and promote intestinal mucosal immune function [14,20]. Furthermore, supplementation with DBSFLM has been reported to enhance intestinal health and increase cecal short-chain fatty acid concentrations in broilers challenged by coccidial infection, suggesting its potential role in gut regulation [21].

Intestinal health is essential for nutrient utilization, growth performance, and disease resistance in poultry [22]. Previous studies have shown that chitin, lauric acid, and antimicrobial substances in BSFLM may promote intestinal health in poultry by improving intestinal villus structure, regulating gut microbiota composition and enhancing intestinal barrier function [6,14,21]. However, research on the application of DBSFLM in indigenous high-quality chicken breeds, especially Wenchang chickens, is still relatively limited, and its comprehensive effects on growth performance, meat quality, and intestinal health remain to be systematically evaluated.

DBSFLM has shown potential as a sustainable protein source in poultry diets, yet its comprehensive effects on slow-growing indigenous chickens remain poorly understood. Accordingly, this study aimed to assess the impact of graded supplementation of DBSFLM in the diet on growth performance, meat quality, antioxidant capacity, intestinal structure, and the cecal microbial community of Wenchang chickens.

## 2. Materials and Methods

### 2.1. Animals and Experimental Design

A total of 90 healthy male Wenchang chickens with similar body weight (1063.33 ± 76.37 g) were purchased from Hainan Tanniu Wenchang Chicken Co., Ltd. (Haikou, China). At 55 days of age, the birds were in the grower period. This age was selected to evaluate the effects of dietary DBSFLM inclusion during the later production stage rather than during the starter phase. After a 5-day adaptation period, the feeding trial commenced at 60 days of age and lasted for 26 days.

The DBSFLM used in this study was obtained as a finished commercial product from a single production batch supplied by Shenzhen Sijifeng Industrial Co., Ltd. (Shenzhen, China). The chickens were randomly allocated to three dietary treatments, with six replicate cages per treatment and five chickens per cage. The basal diet was designated as H0, whereas the experimental diets contained 3% and 5% DBSFLM and were designated as H3 and H5, respectively. Soybean meal was reduced and corn was adjusted accordingly during diet formulation. All diets were formulated according to the Chinese Feeding Standard for Chicken (NY/T 33–2004) [23] to meet the nutrient requirements of growing chickens. Throughout the experiment, the experimental chickens were reared in four-tier cages, with a single cage measuring 70 × 70 × 30 cm, length × width × height, and the temperature, humidity, and lighting conditions of the chicken house were controlled according to standard poultry management requirements. Feeding was offered twice daily at 08:00 and 16:00, and feed and water were supplied ad libitum throughout the trial. Premix and feed ingredients were provided by Hainan New Century Technology Co., Ltd. (Haikou, China). The composition and nutrient levels of the basal diet are shown in Table 1, and the nutritional composition of the DBSFLM, as provided by the manufacturer, is presented in Table 2.

Three experimental diets were formulated: H0, the basal diet without DBSFLM; H3, the diet containing 3% DBSFLM; and H5, the diet containing 5% DBSFLM. DBSFLM was incorporated as a partial replacement of the traditional dietary protein source, with soybean meal reduced as DBSFLM inclusion increased. The proportion of corn was adjusted accordingly to maintain the formulated nutrient specifications of the diets. The dietary treatments were not designed as a simple one-to-one replacement of soybean meal or corn by DBSFLM.

### 2.2. Sample Collection

During the experimental period, feed intake was recorded on a replicate-cage basis. Initial body weight (IBW) and final body weight (FBW) were measured at the beginning and end of the experiment, respectively, after 12 h of feed withdrawal with free access to water. At the end of the experiment, the chickens were fasted for 12 h. Two chickens from each replicate cage were randomly selected for slaughter and sample collection. The two chickens within the same replicate were treated as subsamples, and measurements obtained from the two birds were averaged to generate a single cage-level observation. Therefore, the replicate cage was considered the experimental unit, resulting in six experimental units per treatment (*n* = 6). Immediately after euthanasia (cervical dislocation), breast and leg muscles and abdominal fat samples were collected. A portion of the left breast muscle sample was used for meat-quality evaluation, and the remaining samples were stored at −20 °C for subsequent analysis. During slaughter, jugular vein blood was collected and centrifuged at 3000× *g* for 10 min to obtain serum, which was stored at −20 °C for subsequent analysis. Approximately 5 cm segments of the ileum were collected, rinsed with physiological saline, and fixed in 4% paraformaldehyde for intestinal morphological analysis. Cecal contents were obtained under aseptic conditions, placed in cryogenic vials, and stored at −80 °C for intestinal microbiota analysis.

### 2.3. Growth Performance

The formulas for average daily feed intake (ADFI), average daily gain (ADG), and feed conversion ratio (FCR) were as follows:

ADFI = total feed intake/(experimental period × number of animals)

ADG = total weight gain/(experimental period × number of animals)

FCR = ADFI/ADG

### 2.4. Slaughter Performance

At the end of the experiment, two chickens per replicate were randomly selected, and carcass weight, semi-eviscerated carcass weight, eviscerated carcass weight, breast and leg muscle weights, and abdominal fat weight (abdominal fat including abdominal fat pad and fat surrounding the gizzard) were measured. Slaughter, semi-eviscerated, eviscerated, breast muscle, leg muscle, and abdominal fat percentages were then calculated.

Semi-eviscerated carcass weight: carcass weight with the exclusion of the trachea, esophagus, crop, intestine, spleen, and reproductive organs; retaining heart, lungs, liver (gallbladder removed), kidneys, gizzard, proventriculus (contents and cuticle removed), and abdominal fat.

Eviscerated carcass weight: semi-eviscerated carcass weight excluding heart, lungs, kidneys, proventriculus, gizzard, abdominal fat, head (anterior to the first cervical vertebra), and feet.

Slaughter percentage (%) = (carcass weight/live body weight) × 100

Semi-eviscerated percentage (%) = (semi-eviscerated carcass weight/live body weight) × 100

Eviscerated percentage (%) = (eviscerated carcass weight/live body weight) × 100

Breast muscle percentage (%) = (breast muscle weight/live body weight) × 100

Leg muscle percentage (%) = (leg muscle weight/live body weight) × 100

Abdominal fat percentage (%) = (abdominal fat weight/live body weight) × 100

### 2.5. Meat-Quality Determination

Meat Color and pH Measurement: A colorimeter (CR-10, Konica, Japan) was used to measure lightness (L), redness (a), and yellowness (b*) at three different positions on the right breast muscle, and the average values were calculated.

The pH values of breast muscle were measured using a portable pH meter (testo 205, Testo SE & Co. KGaA, Titisee-Neustadt, Germany) equipped with a penetration pH electrode. The electrode was inserted into three representative locations of the right breast muscle at 45 min and 24 h postmortem, and the three measurements were averaged for each sample.

Drip loss was assessed using breast muscle samples cut to 2.0 cm × 1.0 cm × 0.5 cm. The initial weight of each sample was recorded as m1. Samples were then individually sealed in plastic bags and suspended at 4 °C. After 24 h and 48 h, the samples were reweighed to obtain m2. Drip loss percentage was calculated according to the following formula:

Drip loss (%) = [(m1−m2)/m1] × 100

For cooking yield determination, meat samples were weighed (m1) before heating. The sealed samples were placed in a water bath at 80 °C and maintained for 10 min after the core temperature reached 75 °C. The samples were then cooled to room temperature and reweighed (m2). Cooking yield was calculated as follows:

Cooking yield (%) = (m2/m1) × 100

Shear Force and Subcutaneous Fat Thickness: Fresh breast and leg muscle samples were trimmed into standard strips (1 cm × 1 cm), and shear force was measured using a tenderness meter (C-LM3B, Tenovo International Co., Limited, Beijing, China) with three replicates per sample, and the average value was calculated.

Subcutaneous fat thickness at the tail base was measured using a vernier caliper.

### 2.6. Serum Biochemical Indices

An automatic biochemical analyzer (Hitachi 3500; Hitachi, Tokyo, Japan) was used to quantify serum levels of ALT, AST, ALP, GGT, ALB, TP, UA, BUN, CREA, TBIL, and DBIL. For antioxidant capacity, commercially available assay kits from Nanjing Jiancheng Bioengineering Institute (Nanjing, China) were employed to measure T-AOC, the activities of GSH-Px and T-SOD, as well as MDA concentration in serum.

### 2.7. Intestinal Morphology

After being rinsed with physiological saline, the ileal tissue samples were fixed in 4% paraformaldehyde. After dehydration, embedding, sectioning, and hematoxylin and eosin (H&E) staining, the intestinal tissue morphology was observed under a light microscope (Phenix Optical Co., Ltd., Shangrao, Jiangxi, China). For each section, three fields of view were randomly selected at 40× magnification for photographing and analysis. Using ImageJ 1.54p software (National Institutes of Health, Bethesda, MA, USA), villus height (VH) and crypt depth (CD) were determined, with 5 villi and 5 crypts measured per section, and the villus height to crypt depth ratio (VH/CD) was calculated.

### 2.8. Cecal Microbiota

Cecal content samples were first thawed on ice, mixed thoroughly, and then centrifuged. An aliquot was taken from each sample for microbial DNA extraction with a genomic DNA isolation kit. Integrity of the extracted DNA was checked by 1% agarose gel electrophoresis, while purity and concentration were estimated from the OD260/280 ratio using a NanoDrop 2000 spectrophotometer (Thermo Fisher Scientific, Waltham, MA, USA). The V3–V4 hypervariable region of the bacterial 16S rRNA gene was targeted with primers 338F (5′-ACTCCTACGGGAGGCAGCAG-3′) and 806R (5′-GGACTACHVGGGTWTCTAAT-3′). Following amplification, PCR products were visualized on a 2% agarose gel. Bands of the expected size were excised and purified with an AxyPrep DNA Gel Extraction Kit (Axygen Biosciences, Union City, CA, USA), eluted in Tris-HCl buffer, and quantified using a QuantiFluor™-ST fluorometer (Promega Corporation, Madison, WI, USA). Purified amplicons were then used to prepare a sequencing library. Libraries that passed quality control were sequenced on an Illumina MiSeq PE300 platform (Illumina, San Diego, CA, USA). DNA extraction and sequencing were performed by Shanghai Majorbio Bio-pharm Technology Co., Ltd. (Shanghai, China).

The Majorbio Cloud Platform (Majorbio Bio-pharm Technology Co., Ltd., Shanghai, China) was employed for all sequencing data processing. Raw reads were merged and quality-filtered to obtain high-quality sequences. Using USEARCH (version 11.0.667, drive5, Tiburon, CA, USA), these sequences were clustered into operational taxonomic units (OTUs) at a 97% similarity threshold. For taxonomic assignment, OTU representative sequences were aligned against the SILVA database (version 138, Max Planck Institute for Marine Microbiology, Bremen, Germany) with the RDP Classifier. Alpha diversity was quantified by calculating the Shannon index and the Chao1 richness estimator. Beta diversity patterns were visualized through principal coordinate analysis (PCoA) based on Bray–Curtis distances, and differences in microbial community structure among treatments were tested using permutational multivariate analysis of variance (PERMANOVA). Linear discriminant analysis effect size (LEfSe) was then applied to identify differentially abundant bacterial taxa among groups, using a linear discriminant analysis (LDA) score threshold of 2.5.

### 2.9. Data Analysis

Raw data were first organized in Microsoft Excel 2021. Statistical analyses were subsequently conducted using IBM SPSS Statistics 26.0 (IBM Corp., Armonk, NY, USA). All values are presented as mean ± standard deviation. The replicate cage was considered the experimental unit for all statistical analyses. For variables measured from two chickens per cage, the two measurements were averaged within each cage before statistical analysis, resulting in six experimental units per treatment. Treatment effects were evaluated using one-way analysis of variance (ANOVA), followed by Tukey’s multiple-comparison test when the overall treatment effect was significant. Normality and homogeneity of variance were assessed before analysis. Graphs were generated using GraphPad Prism 10.0 (GraphPad Software Inc., San Diego, CA, USA). In the figures, statistical significance is indicated by asterisks: * *p* < 0.05, ** *p* < 0.01, *** *p* < 0.001, **** *p* < 0.0001.

## 3. Results

### 3.1. Growth Performance

Table 3 shows that there were no significant differences in IBW of Wenchang chickens among all groups at the start of the experiment. Dietary supplementation with DBSFLM exerted no significant effects on FBW, ADFI, ADG, and FCR of Wenchang chickens.

### 3.2. Slaughter Performance

Replacing conventional protein sources with DBSFLM in the diet did not significantly alter slaughter yield, semi-eviscerated yield, eviscerated yield, breast muscle yield, leg muscle yield, or subcutaneous fat thickness in Wenchang chickens (Table 4). However, abdominal fat percentage responded differently: birds receiving the H5 diet exhibited a significantly higher value than those fed the control diet (*p* = 0.033). The H3 group showed an intermediate response, with abdominal fat percentage not differing significantly from either the control or H5 group.

### 3.3. Meat Quality

As presented in Table 5, replacement with DBSFLM had no significant influence on muscle pH, drip loss, cooking yield, shear force, or color parameters (L*, a*, b*) of Wenchang chickens. Nevertheless, the H5 group showed a tendency toward a lower redness value (a*) compared with the control group, although the difference was not statistically significant.

### 3.4. Antioxidant Capacity

**Figure 1:**
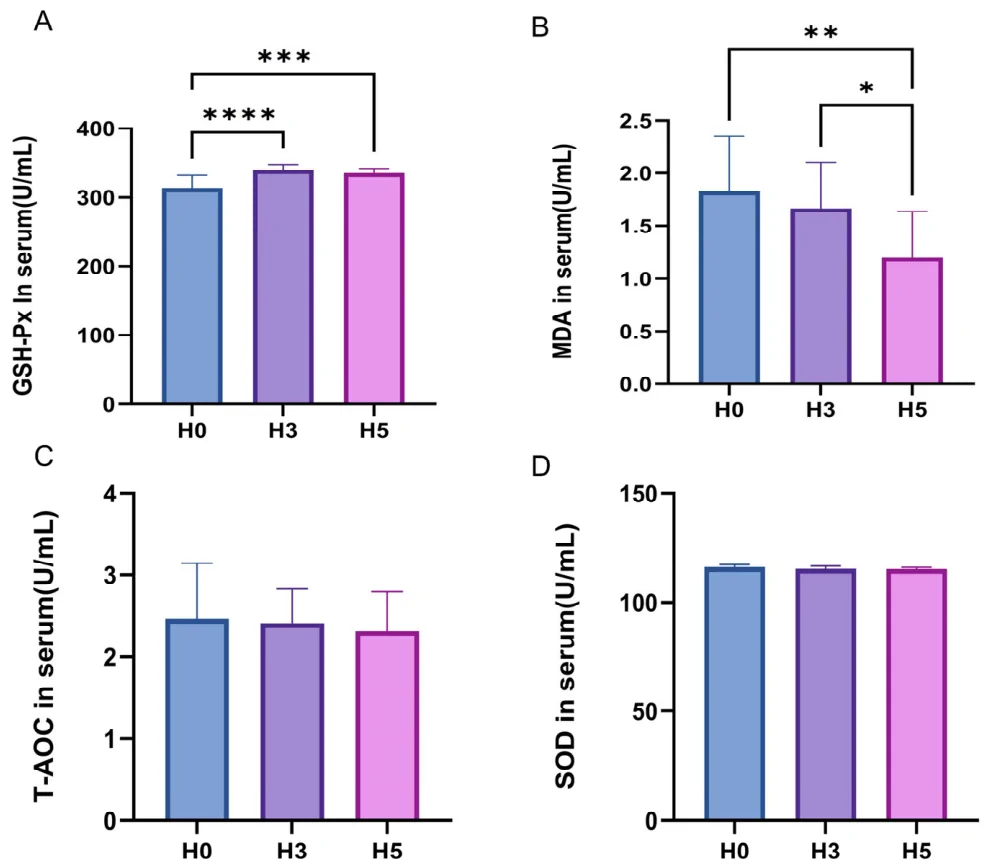
Effects of dietary defatted black soldier fly larvae meal on serum antioxidant indices in Wenchang chickens (*n* = 6). (**A**) Glutathione peroxidase (GSH-Px); (**B**) Malondialdehyde (MDA); (**C**) Total antioxidant capacity (T-AOC); (**D**) Total superoxide dismutase (T-SOD). * *p* < 0.05, ** *p* < 0.01, *** *p* < 0.001, **** *p* < 0.0001.

Serum T-SOD activity and T-AOC did not differ significantly among the experimental groups. However, replacing conventional protein sources with DBSFLM led to marked changes in GSH-Px activity and MDA content, as illustrated in Figure 1. Relative to H0, GSH-Px activity in serum was significantly elevated in the H3 and H5 groups (*p* < 0.05). Serum MDA concentration in the H5 group was also significantly lower than in both the H0 and H3 groups (*p* < 0.05).

### 3.5. Serum Biochemistry

As shown in Table 6, compared with the control group, serum CREA levels were significantly lower in the H3 and H5 groups (*p* = 0.003). Although reductions in ALT and AST activities were observed in the H3 group, these changes did not reach statistical significance. Furthermore, dietary treatments had no significant effects on ALB, TP, TBIL, DBIL, UA, GGT, BUN or ALP concentrations.

### 3.6. Intestinal Morphology

**Figure 2:**
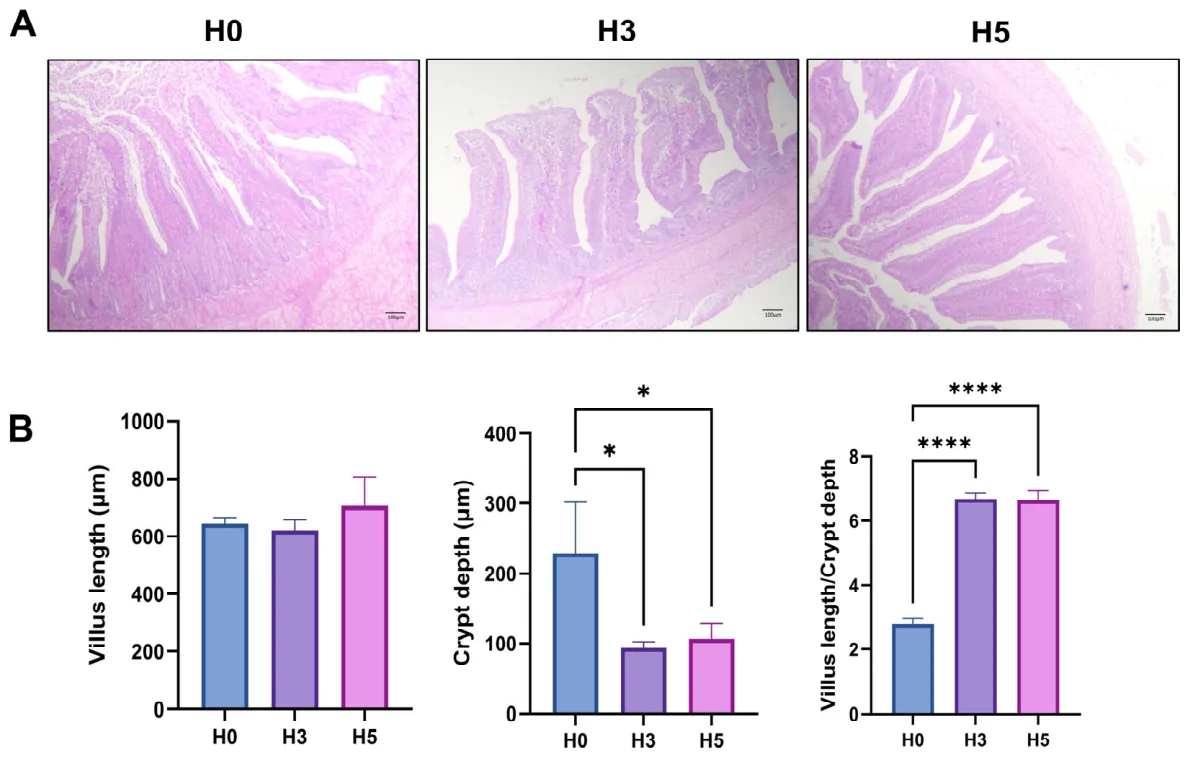
Effects of dietary DBSFLM supplementation on ileal morphology of Wenchang chickens presented by hematoxylin and eosin (H&E) staining (×40) (*n* = 6); (**A**) Microscope scanning; (**B**) Villus height (VH), Crypt depth (CD), Villus height/Crypt depth (VH/CD); Data are presented as means ± SD. * *p* < 0.05, **** *p* < 0.0001.

Figure 2 presents the results for ileal morphology across dietary treatments. A significant reduction in crypt depth was observed in the H3 and H5 groups relative to H0 (*p* < 0.05). The VH/CD also increased significantly in both groups (*p* < 0.01). Villus height in H5 was numerically greater than in controls, but this difference failed to reach statistical significance.

### 3.7. Gut Microbiota

**Figure 3:**
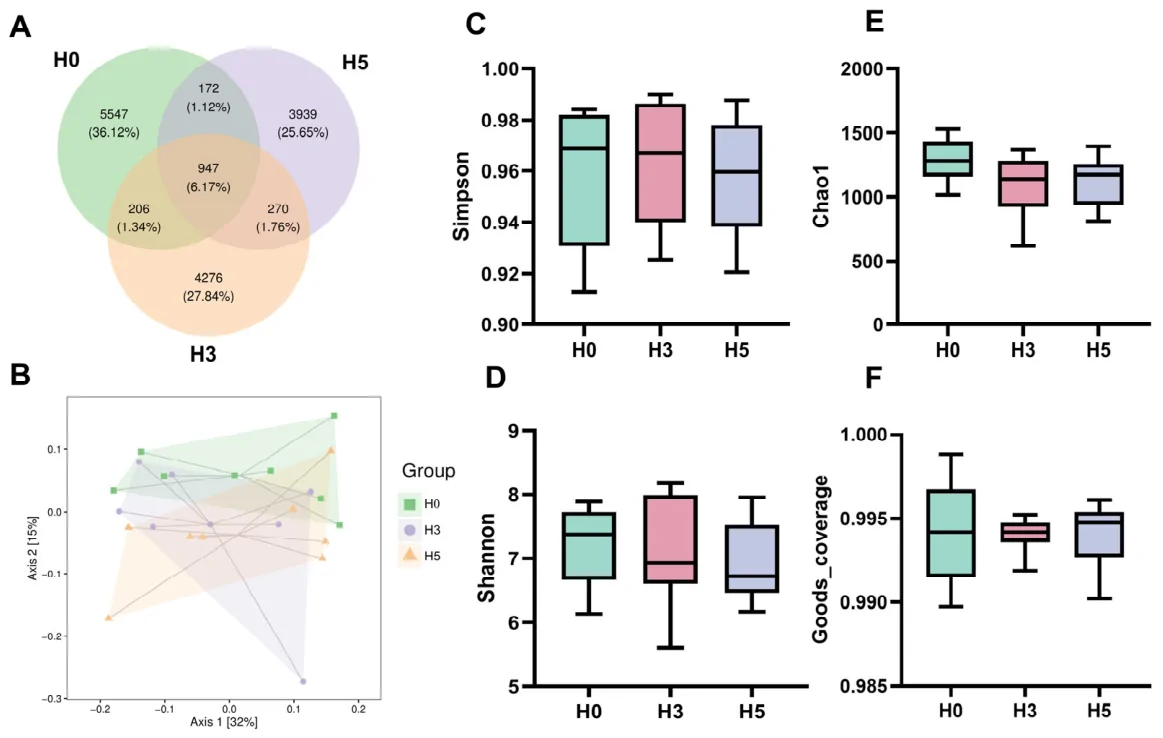
Effects of dietary DBSFLM supplementation on cecal microbiota of Wenchang chickens (*n* = 6). (**A**) OTU Venn diagram; (**B**) Principal coordinate analysis (PCoA) of gut microbial structure; (**C**) Simpson index; (**D**) Shannon index; (**E**) Chao1 index; (**F**) Goods_coverage index. Data are presented as means ± SD.

**Figure 4:**
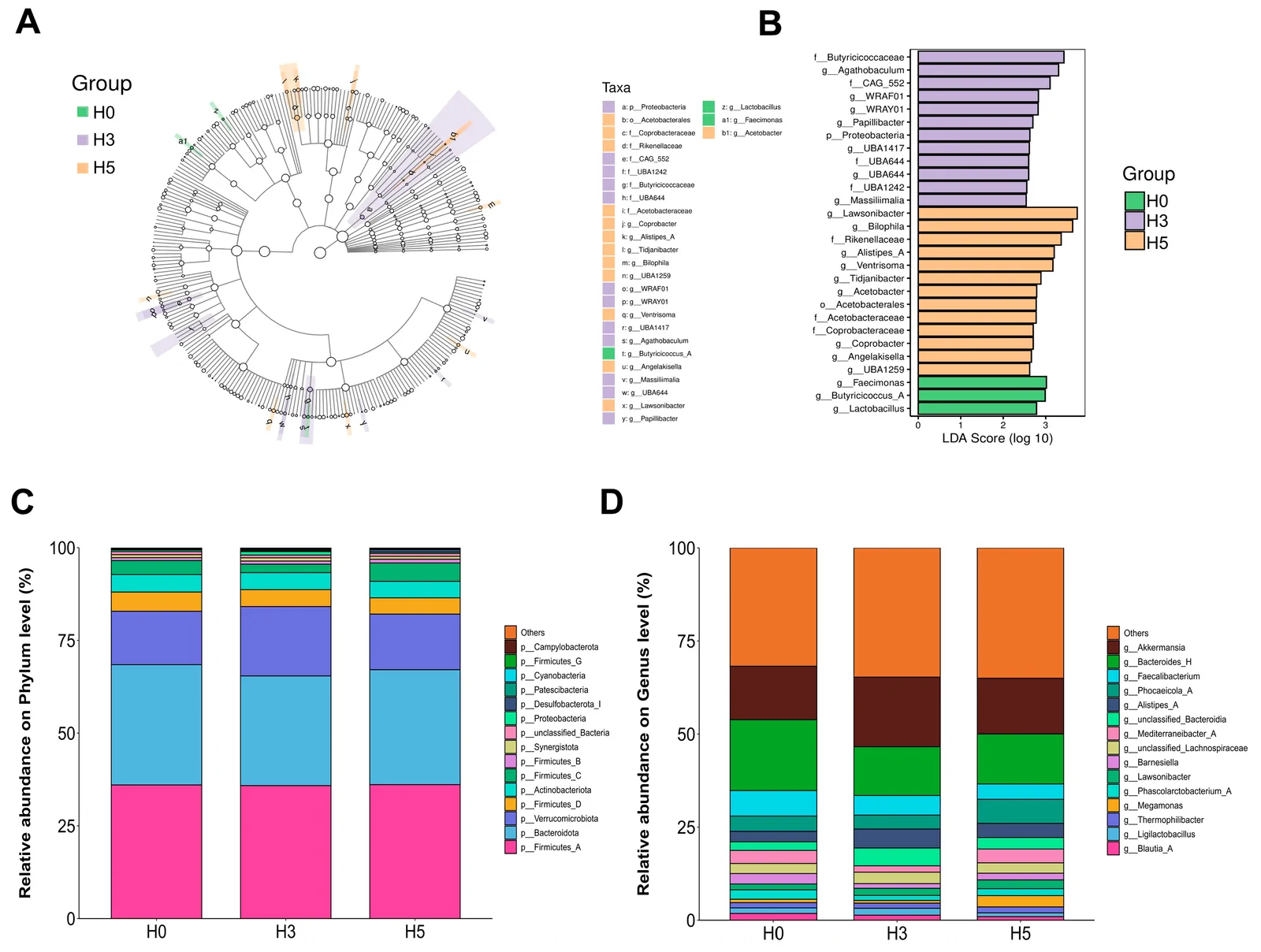
Effects of dietary treatment on the cecal bacterial relative abundance and dominant microbiota of Wenchang chickens (*n* = 6). (**A**) Discriminant analysis effect size (LEfSe) analysis cladogram—The cladogram illustrates the hierarchical taxonomic relationships among the identified microbial taxa, ranging from phylum to genus level. Circles radiating from the innermost to the outermost layers represent taxonomic levels in descending order: phylum (a), order (b), family (c–i), and genus (j–y). The diameter of each node is proportional to the relative abundance of the corresponding taxon; (**B**) Linear Discriminant Analysis (LDA) effect size plot—bar length corresponds to LDA score, and only taxa with LDA score > 2.5 are shown; (**C**) Relative abundance of microbial composition at the phylum level; (**D**) Relative abundance of microbial composition at the genus level.

The influence of DBSFLM on cecal microbial composition is presented in Figure 3 and Figure 4. Venn analysis showed 206 OTUs shared between H0 and H3, 172 OTUs shared between H0 and H5, 270 OTUs shared between H3 and H5, and 947 OTUs shared among all three treatment groups, corresponding to 1.34%, 1.12%, 1.76%, and 6.17% of the total OTUs, respectively. The numbers of unique OTUs were 5547, 3939, and 4276 for H0, H5, and H3, corresponding to 36.12%, 25.65%, and 27.84%, respectively, indicating variation in cecal microbial composition among treatments.

Bray–Curtis dissimilarity-based PCoA revealed a partial separation of microbial community structure among treatments; however, this separation was not statistically significant.

Cecal microbiota alpha diversity was assessed using multiple indices. No significant differences among the treatment groups were detected for the Shannon diversity index, Simpson diversity index, Chao1 index, or Good’s coverage index. These results indicate that DBSFLM supplementation did not markedly alter overall microbial diversity or sequencing coverage. Nevertheless, a numerical trend was observed: the H5 group exhibited higher Shannon and Chao1 indices than the control group, although this difference did not reach statistical significance.

Differential bacterial taxa were identified among the dietary treatments using LEfSe analysis. Lactobacillus and *Faecimonas* were more abundant in the H0 group, whereas the H3 group exhibited higher relative abundances of Butyricicoccaceae, *Agathobaculum*, and *Papillibacter*. Firmicutes_A, Bacteroidota, Verrucomicrobiota, and Proteobacteria were the dominant phyla in all groups. Replacing conventional protein sources with DBSFLM increased the relative abundance of Firmicutes_A compared with the H0 group, while the H5 group showed reduced relative abundances of Bacteroidota and Proteobacteria. *Akkermansia*, *Bacteroides*, *Ruminococcus*, and *Faecalibacterium* represented the predominant genera. In comparison with the control group, higher relative abundances of *Akkermansia* and lower abundances of *Bacteroides* were observed in the groups receiving DBSFLM-containing diets.

## 4. Discussion

No statistically significant differences were observed in average daily feed intake, average daily gain, or feed conversion ratio among the dietary treatments. Similar observations have been reported in broilers receiving appropriate levels of BSFLM, in which growth performance and feed conversion ratio were not adversely affected [6]. The comparable growth performance observed in the present study suggests that partial replacement of traditional dietary protein sources with 3% or 5% DBSFLM provided sufficient nutrients to support normal growth during the experimental period. The relatively high protein content and favorable amino acid characteristics of black soldier fly larvae meal may contribute to its suitability as a protein ingredient [5]. In addition, defatting can increase the relative protein concentration of the meal and may affect its nutrient utilization characteristics [20].

Most carcass traits were also not significantly different among treatments. However, abdominal fat percentage was significantly higher in the H5 group than in the H0 group. Similar effects of insect-derived dietary ingredients on carcass fat deposition have been reported in poultry [24,25,26]. The increase in abdominal fat at the higher DBSFLM inclusion level may reflect differences in nutrient partitioning associated with dietary treatment. Previous studies have indicated that insect-derived lipids, including medium-chain fatty acids such as lauric acid, can influence lipid metabolism and energy utilization in poultry [27]. Nevertheless, the present study did not experimentally determine the complete fatty acid composition or metabolizable energy of the DBSFLM used in the diets. Therefore, the specific nutritional factor responsible for the increased abdominal fat deposition cannot be established from the present data. Importantly, the increase in abdominal fat was not accompanied by statistically significant changes in slaughter percentage, eviscerated percentage, breast muscle percentage, or leg muscle percentage, suggesting that the observed response was relatively specific to abdominal fat deposition under the conditions tested.

Dietary DBSFLM was not associated with statistically significant differences in muscle pH, drip loss, cooking yield, shear force, or meat color. Similar results have been reported in poultry receiving insect-derived dietary ingredients, in which appropriate inclusion levels did not markedly alter the evaluated meat quality characteristics [28,29]. The relatively stable pH and water-related meat quality parameters among treatments suggest that partial replacement of traditional dietary protein sources with DBSFLM did not result in detectable changes in the measured postmortem meat quality characteristics during the experimental period.

The numerical decrease in a* value observed in the H5 group did not reach statistical significance. Overall, the present results indicate that, under the dietary and experimental conditions evaluated, DBSFLM was not associated with statistically significant changes in the measured meat quality traits.

Serum biochemical parameters provide useful information regarding physiological and metabolic status. In the present study, dietary DBSFLM significantly decreased serum creatinine concentration, whereas ALT and AST showed numerical decreasing trends without statistically significant differences. Previous research has reported that BSFLM inclusion did not adversely affect serum biochemical indicators related to liver function in broilers [6]. Together, these observations indicate that the DBSFLM inclusion levels evaluated in the present study were not associated with detectable adverse changes in the measured serum biochemical parameters.

In addition to the biochemical parameters, dietary DBSFLM was associated with increased serum GSH-Px activity and decreased MDA concentration. GSH-Px is an important component of the enzymatic antioxidant defense system, whereas MDA is commonly used as an indicator of lipid peroxidation. Under normal physiological conditions, reactive oxygen species are continuously generated through metabolic processes, and endogenous antioxidant systems contribute to maintaining redox homeostasis [30,31]. Therefore, the changes observed in GSH-Px activity and MDA concentration indicate alterations in serum antioxidant-related parameters and lipid peroxidation status following dietary DBSFLM inclusion.

The mechanisms responsible for these changes remain unclear. Black soldier fly-derived ingredients contain several nutritional and potentially bioactive components, including chitin-related compounds, antimicrobial peptides, and functional fatty acids [2,32]. Previous studies have discussed the potential biological activities of chitin-derived compounds and insect-derived lipids [33,34]. However, the present study did not comprehensively characterize the amino acid, fatty acid, mineral, peptide, or other bioactive components of the DBSFLM batch used in the experiment. Therefore, the observed changes in GSH-Px and MDA cannot be attributed to any specific component of DBSFLM. Further studies combining detailed DBSFLM compositional analysis with molecular markers of antioxidant signaling would be required to clarify the underlying mechanisms.

Dietary DBSFLM significantly decreased ileal crypt depth and increased the villus height-to-crypt depth ratio. Intestinal morphology is an important structural characteristic of the intestinal tract and can provide information about changes in the intestinal mucosal architecture [35,36]. Similar changes in intestinal morphology have been reported in poultry receiving insect-derived dietary ingredients [36].

In the present study, the reduced crypt depth and increased VH/CD ratio indicate changes in ileal morphological characteristics following DBSFLM inclusion. However, these morphological observations alone do not directly demonstrate improved nutrient digestibility, intestinal barrier function, or absorptive capacity. Although previous studies have discussed relationships between intestinal morphology and intestinal physiological function [35,37,38], the present study did not directly measure nutrient digestibility, intestinal permeability, tight-junction proteins, or inflammatory responses. Therefore, the observed morphological changes should be interpreted as structural responses rather than definitive evidence of improved overall intestinal health.

The biological mechanisms underlying these morphological changes remain uncertain. Insect-derived feed ingredients contain structural components such as chitin that may interact with the gastrointestinal environment and intestinal microbial community [32,33].

Dietary DBSFLM was associated with changes in cecal microbial composition, whereas no statistically significant differences were observed in the measured alpha-diversity indices. Similar observations have been reported in studies of insect-derived feed ingredients, in which changes in specific bacterial taxa occurred without marked alterations in overall microbial diversity [38,39]. This suggests that dietary DBSFLM may influence the relative distribution of particular bacterial taxa rather than causing a broad alteration in overall microbial diversity.

At the phylum level, DBSFLM-containing diets were associated with an increased relative abundance of Firmicutes_A and a decreased relative abundance of Proteobacteria. Firmicutes include bacterial groups involved in the fermentation of dietary substrates and production of microbial metabolites, including short-chain fatty acids [40].

Proteobacteria are commonly present in the intestinal microbiota, and changes in their relative abundance have been discussed in relation to intestinal microbial imbalance and inflammatory conditions [41]. Therefore, the lower relative abundance of Proteobacteria observed in the H5 group may indicate a shift in microbial community structure.

At the genus level, higher relative abundances of *Akkermansia* were observed in the DBSFLM-containing groups. *Akkermansia* has been studied in relation to mucin utilization and host–microbe interactions and has been associated with intestinal barrier and metabolic functions [42]. Thus, the change in *Akkermansia* observed in the present study may be biologically relevant.

The relative abundances of Butyricicoccaceae and *Agathobaculum* also differed among dietary treatments. These taxa are of interest in the context of intestinal microbial metabolism, particularly microbial fermentation and short-chain fatty acid production. Butyrate itself can serve as an important energy source for intestinal epithelial cells and is involved in the regulation of intestinal barrier and immune functions [43].

Taken together, the microbiota results indicate that partial replacement of traditional dietary protein sources with DBSFLM was associated with changes in the relative abundance of several bacterial taxa without a marked alteration in overall alpha diversity. These microbial changes occurred concurrently with changes in serum antioxidant-related parameters and ileal morphology. However, the present experimental design does not permit causal relationships to be established between microbial composition and these physiological responses. Future studies integrating microbial metabolites, intestinal barrier markers, inflammatory indicators, and nutrient digestibility measurements would be valuable for determining whether the observed microbial changes have functional consequences for intestinal physiology.

A limitation of the present study is the lack of a detailed characterization of DBSFLM, including its amino acid profile, fatty acid composition, and other functional components. Considering that these nutritional components may influence nutrient deposition and meat quality formation, future studies should investigate the effects of DBSFLM supplementation during the finishing stage of Wenchang chickens to clarify its potential regulatory roles in muscle development, lipid metabolism, and meat-quality improvement.

## 5. Conclusions

Under the conditions of the present study, dietary inclusion of 3% or 5% DBSFLM as a partial replacement of traditional protein sources did not result in statistically significant differences in growth performance or most of the measured meat quality traits in Wenchang chickens. However, 5% DBSFLM increased abdominal fat percentage, while dietary DBSFLM was associated with changes in serum antioxidant-related parameters, including increased GSH-Px activity and decreased MDA concentration. In addition, DBSFLM inclusion was associated with changes in ileal morphology and cecal microbial composition, including alterations in the relative abundances of several bacterial taxa. These findings suggest that DBSFLM may influence systemic oxidative status and intestinal characteristics in growing Wenchang chickens; however, the underlying mechanisms cannot be established from the present data. Further studies integrating intestinal barrier function, inflammatory responses, nutrient digestibility, microbial metabolites, short-chain fatty acids, and comprehensive characterization of DBSFLM composition are warranted. Environmental and economic benefits of DBSFLM should also be evaluated using dedicated life-cycle, environmental, and economic assessments.

## Figures and Tables

**Table 1:** Feed formulation and nutrient levels.

		Diet ^1^	
Ingredient	H0	H3	H5
Dietary composition (% of diet, DM basis)			
Corn	65.74	66.47	66.96
Soybean meal	16.94	13.49	11.03
Wheat bran	10.02	10.04	10.01
Dicalcium phosphate (dihydrate)	2.60	2.60	2.60
Salt	0.40	0.40	0.40
Limestone	2.30	2.00	2.00
DBSFLM	0.00	3.00	5.00
Premix ^2^	2.00	2.00	2.00
Total	100	100	100
Nutrient composition (% of DM)			
CP (%)	15.87	15.93	15.90
EE (%)	2.60	2.88	3.07
CF (%)	2.36	2.24	2.15
ME (MJ/kg)	11.13	11.25	11.31
Ash (%)	2.11	1.99	1.90
Ca (%)	1.51	1.47	1.52
P (%)	0.84	0.85	0.85
D-Lys (%)	0.72	0.70	0.69
D-Met (%)	0.25	0.25	0.25
Na (%)	0.18	0.18	0.18
Cl (%)	0.28	0.28	0.28

^1^ Experimental diets: H0: basal diet without DBSFLM; H3: diet containing 3% DBSFLM; H5: diet containing 5% DBSFLM. DBSFLM was included as a partial replacement of the traditional dietary protein source. ^2^ Premix provided per kilogram of diet: vitamin A, 2700 IU/kg; vitamin D, 400 IU/kg; vitamin E, 10 IU/kg; vitamin K, 0.5 mg/kg; thiamin, 2 mg/kg; riboflavin, 5 mg/kg; niacin, 30 mg/kg; pantothenic acid, 10 mg/kg; pyridoxine, 3 mg/kg; biotin, 0.1 mg/kg; folic acid, 0.5 mg/kg; vitamin B12, 0.01 mg/kg; choline, 750 mg/kg; linoleic acid, 1%; Fe, 80.00 mg/kg; Cu, 8.00 mg/kg; Mn, 80.00 mg/kg; Zn, 80.00 mg/kg; I, 0.70 mg/kg; Se, 0.30 mg/kg. Note: Amino acids were calculated values, while the others were measured values.

**Table 2:** Composition of DBSFLM.

Item	Content (%)
CP (%)	54.93
EE (%)	9.92
Ash (%)	1.38
CF (%)	0.29
Ca (%)	2.82
P (%)	0.79
D-Lys (%)	2.86
D-Met (%)	0.78
ME (MJ/kg)	12.1

**Table 3:** Effects of dietary DBSFLM inclusion as a partial replacement of traditional protein sources on the growth performance of Wenchang chickens.

Item	H0	H3	H5	*p*-Value
IBW (g)	1056 ± 70.55	1068 ± 81.20	1062 ± 90.67	0.985
FBW (g)	1599 ± 68.47	1608 ± 65.80	1614 ± 83.65	0.942
ADFI (g)	87.31 ± 5.01	89.34 ± 2.18	86.31 ± 3.87	0.407
ADG (g)	20.76 ± 0.77	20.78 ± 0.68	21.22 ± 1.12	0.607
FCR	4.21 ± 0.21	4.30 ± 0.15	4.08 ± 0.35	0.354

H0: basal diet without defatted black soldier fly larvae meal (DBSFLM); H3: diet containing 3% DBSFLM; H5: diet containing 5% DBSFLM. Values are presented as mean ± SD (*n* = 6 replicate cages per treatment).

**Table 4:** Effects of dietary DBSFLM inclusion as a partial replacement of traditional protein sources on the slaughter performance of Wenchang chickens.

Item	H0	H3	H5	*p*-Value
Slaughter percentage (%)	89.17 ± 0.96	89.82 ± 0.59	88.93 ± 1.58	0.386
Semi-eviscerated percentage (%)	81.79 ± 0.89	82.09 ± 1.09	81.77 ± 1.03	0.831
Eviscerated percentage (%)	66.04 ± 1.14	65.45 ± 1.74	65.76 ± 1.32	0.773
Breast muscle percentage (%)	12.99 ± 0.38	12.42 ± 0.55	13.11 ± 0.63	0.085
Leg muscle percentage (%)	24.82 ± 1.77	24.35 ± 1.04	23.37 ± 1.33	0.226
Abdominal fat percentage (%)	0.78 ± 0.35 ^a^	1.22 ± 0.58 ^ab^	1.68 ± 0.62 ^b^	0.033
Subcutaneous fat thickness (mm)	2.85 ± 0.36	2.89 ± 0.15	3.15 ± 0.35	0.206

Note: Different lowercase letters in the same row indicate significant differences (*n* = 6).

**Table 5:** Effects of dietary DBSFLM inclusion as a partial replacement of traditional protein sources on the meat quality of Wenchang chickens.

Item	H0	H3	H5	*p*-Value
pH_45 min_	6.25 ± 0.10	6.22 ± 0.15	6.15 ± 0.10	0.350
pH_24 h_	6.14 ± 0.21	5.99 ± 0.16	6.06 ± 0.14	0.364
Drip loss at 24 h (%)	4.67 ± 2.39	3.71 ± 0.59	3.70 ± 0.82	0.448
Drip loss at 48 h (%)	7.12 ± 3.08	7.36 ± 0.69	7.22 ± 1.34	0.978
Cooking yield (%)	72.71 ± 2.00	72.44 ± 2.87	72.18 ± 0.92	0.908
Shear force (N)	53.94 ± 17.45	55.52 ± 18.77	48.20 ± 10.46	0.711
Lightness (L*)	51.65 ± 3.61	52.93 ± 1.97	54.22 ± 1.67	0.252
Redness (a*)	7.88 ± 1.94	7.32 ± 1.23	6.12 ± 1.17	0.146
Yellowness (b*)	8.13 ± 1.60	8.72 ± 1.40	9.70 ± 1.57	0.229

**Table 6:** Effects of dietary DBSFLM inclusion as a partial replacement of traditional protein sources on serum biochemical indices of Wenchang chickens.

Item	H0	H3	H5	*p*-Value
CREA (μmol/L)	33.00 ± 5.14 ^a^	18.57 ± 4.13 ^b^	20.75 ± 8.96 ^b^	0.003
BUN (mmol/L)	1.32 ± 0.11	1.28 ± 0.07	1.35 ± 0.11	0.501
ALB (g/L)	13.23 ± 0.57	12.34 ± 2.91	12.88 ± 0.70	0.684
TP (g/L)	42.07 ± 9.44	36.29 ± 8.28	39.93 ± 7.89	0.513
TBIL (μmol/L)	21.81 ± 5.30	27.23 ± 10.08	26.48 ± 3.73	0.365
ALT (U/L)	2.58 ± 1.28	2.17 ± 0.88	3.83 ± 2.54	0.249
AST (U/L)	402.83 ± 76.99	370.83 ± 71.79	484.42 ± 108.00	0.099
GGT (U/L)	25.58 ± 1.69	23.08 ± 3.98	23.33 ± 5.37	0.504
UA (μmol/L)	335.25 ± 60.85	260.42 ± 50.04	302.08 ± 56.21	0.099
DBIL (μmol/L)	4.53 ± 2.87	5.66 ± 2.04	6.55 ± 0.97	0.282
ALP (U/L)	380.83 ± 109.94	354.58 ± 141.82	508.17 ± 189.38	0.202

Note: Different lowercase letters in the same row indicate significant differences (*n* = 6).

## Data Availability

The raw sequencing data generated in this study have been deposited in the National Center for Biotechnology Information (NCBI) Sequence Read Archive (SRA) under BioProject accession number PRJNA1505176. The data that support the findings of this study are available from the corresponding authors upon reasonable request.

## Abbreviations

The following abbreviations are used in this manuscript:

DBSFLM	defatted black soldier fly larvae meal
BSFLM	black soldier fly larvae meal
IBW	initial body weight
FBW	final body weight
ADFI	average daily feed intake
ADG	average daily gain
FCR	feed conversion ratio
GSH-Px	glutathione peroxidase
MDA	malondialdehyde
T-AOC	total antioxidant capacity
T-SOD	total superoxide dismutase
CD	crypt depth
VH	villus height
VH/CD	villus height to crypt depth ratio
ALT	alanine aminotransferase
AST	aspartate aminotransferase
ALP	alkaline phosphatase
GGT	γ-glutamyl transpeptidase
ALB	albumin
TP	total protein
UA	uric acid
BUN	blood urea nitrogen
TBIL	total bilirubin
DBIL	direct bilirubin
CREA	creatinine
H&E	hematoxylin and eosin
OTUs	operational taxonomic units
PCoA	principal coordinate analysis
LEfSe	linear discriminant analysis effect size
LDA	linear discriminant analysis
